# miRNA-7a-2-3p Inhibits Neuronal Apoptosis in Oxygen-Glucose Deprivation (OGD) Model

**DOI:** 10.3389/fnins.2019.00016

**Published:** 2019-01-23

**Authors:** Zi-Bin Zhang, Ya-Xin Tan, Qiong Zhao, Liu-Lin Xiong, Jia Liu, Fei-Fei Xu, Yang Xu, Larisa Bobrovskaya, Xin-Fu Zhou, Ting-Hua Wang

**Affiliations:** ^1^Institute of Neuroscience, Kunming Medical University, Kunming, China; ^2^Department of Anesthesiology, Qilu Hospital of Shandong University, Jinan, China; ^3^Department of Anesthesiology, Sun Yat-sen Memorial Hospital, Sun Yat-sen University, Guangzhou, China; ^4^School of Pharmacy and Medical Sciences, Faculty of Health Sciences, University of South Australia, Adelaide, SA, Australia; ^5^Department of Laboratory Animal Science, School of Basic Medical Sciences, Kunming Medical University, Kunming, China; ^6^Institute of Neurological Diseases, Department of Anesthesiology and Translational Neuroscience Center, West China Hospital, Sichuan University, Chengdu, China

**Keywords:** neonatal hypoxic-ischemic brain damage (HIBD), miR-7a-2-3p, oxygen-glucose deprivation (OGD), neuronal apoptosis, Vimentin (VIM)

## Abstract

Neuronal apoptosis is a major pathological hallmark of the neonatal hypoxic-ischemic brain damage (HIBD); however, the role of miR-7a-2-3p in the regulation of HIBD remains unknown. The purpose of this study was to explore the possible roles of miR-7a-2-3p in brain injury using a hypoxia-ischemia model in rats and oxygen-glucose deprivation (OGD) model *in vitro*. Firstly, we established the hypoxia-ischemia (HI) model and verified the model using Zea Longa scores and MRI in rats. Next, the changes of miR-7a-2-3p were screened in the ischemic cortex of neonatal rats by qRT-PCR at 12, 48, and 96 h after HIBD. We have found that the expression of miR-7a-2-3p in the HI rats decreased significantly, compared with the sham group (*P* < 0.01). Then, we established the OGD model in PC12 cells, SH-SY5Y cells and primary cortical neurons *in vitro* and qRT-PCR was used to confirm the changes of miR-7a-2-3p in these cells after the OGD. In order to determine the function of miR-7a-2-3p, PC12 cells, SH-SY5Y cells and rat primary cortical neurons were randomly divided into normal, OGD, mimic negative control (mimic-NC) and miR-7a-2-3p groups. Then, Tuj1+ (neuronal marker) staining, TUNEL assay (to detect apoptotic cells) and MTT assay (to investigate cell viability) were performed. We have found that the number of PC12 cells, SH-SY5Y cells and cortical neurons in the miR-7a-2-3p groups increased significantly (*P* < 0.01) in comparison to the OGD groups. The survival of cortical neurons in the miR-7a-2-3p group was improved markedly (*P* < 0.01), while the apoptosis of neurons in the miR-7a-2-3p group was significantly decreased (*P* < 0.01), compared with the normal group. Lastly, we investigated the target genes of miR-7a-2-3p by using the prediction databases (miRDB, TargetScan, miRWalk, and miRmap) and verified the target genes with qRT-PCR in the HI rats. Bioinformatics prediction showed that Vimentin (VIM), pleiomorphic adenoma gene 1(PLAG1), dual specificity phosphatase 10 (DUSP10), NAD(P)H dehydrogenase, quinone 1 (NQO1) and tumor necrosis factor receptor superfamily member 1B (TNFRSF1B) might be the targets of miR-7a-2-3p and the qRT-PCR confirmed that VIM increased in the HI rats (*P* < 0.01). In conclusion, miR-7a-2-3p plays a crucial role in the hypoxic-ischemic injury, and is associated with regulation of VIM.

## Introduction

Neonatal ischemic hypoxic brain damage (HIBD) is caused by intrauterine asphyxia and congenital diseases and is common in neonates of perinatal period (Fenichel, 1983; DelRosso et al., 2017). Furthermore, neonatal HIBD not only threatens the life of neonates, but also leads to series of secondary neuronal damages after perinatal period (Gieron-Korthals and Colon, 2005), which are related to remote organ dysfunctions (Klempt et al., 1992; Perlman, 2006). However, the mechanisms of neonatal HIBD are not entirely clear and the effective treatment strategy is not identified, therefore it is important to explore the molecular mechanisms of HIBD.

MicroRNAs (miRNAs) are a class of non-coding RNAs, approximately 20–22 nucleotides in length, which are involved in regulation of gene expression at the post-transcriptional level (Moss, 2002; Shi, 2003; Bartel, 2004). MiRNAs can bind to the 3’untranslated region (3’UTR) of a target gene, resulting in the target mRNA degradation or translational repression (Bagga et al., 2005; Nohata et al., 2011), which are involved in diverse biological processes, such as cell proliferation, apoptosis and death (Ambros, 2004; Li et al., 2009; Fasanaro et al., 2010; Son et al., 2014). In the recent years, researchers have found that miRNAs are related to nerve repair and regeneration closely (Choi et al., 2015). It has been shown that mature miR-7 is regulated by the transcription factor HoxD10 (Reddy et al., 2008). Furthermore, researchers have found that miR-7 is highly connected to cancerous cell proliferation and metastasis, visual abnormalities, myocardial fibrosis and neuropathic pain (Sakai et al., 2013). However, the role of miR-7a-2-3p in hypoxic-ischemic brain damage is unclear. Therefore, it is important to explore the molecular effects of miR-7a-2-3p in HIBD.

Thus, our study aimed to investigate the possible effects of miR-7a-2-3p on the hypoxic ischemic neurons in the neonatal HIBD. Investigation of these mechanisms could provide a novel treatment strategy for neonatal HIBD.

## Materials and Methods

### Animals Care and Grouping

Neonatal rats (seven-day old Sprague-Dawley (SD) rats) were provided by Experimental Animal Center of Kunming Medical University (China). The animal study protocol was approved by the Animal Care and Welfare Committee of Kunming Medical University with the approval number: 2015-1A. All experiments conformed to the Guides for the Care and Use of Laboratory Animals published by the US National Institutes of Health. The rats were divided randomly into a sham group and hypoxia-ischemia (HI) group. Animals were resided in cages with a 12-h light/dark cycle and could freely obtain food and water. Aseptic environment was maintained during surgical procedures.

### Establishing the HI Model in Rats

We established the HI model according to the previously validated model by Rice et al. (1981). Before the operation, the seven-day-postnatal rats were anesthetized with isoflurane. The midline of the ventral cervical skin was incised following the blunt dissection of parenchyma and the right common carotid artery was exposed. Then, the right common carotid artery was ligated by electric coagulating apparatus. Finally, the skin was closed and stitched and the newborn rats were allowed to recover for 1 h. Then the rats were placed into the anoxic box with 8% oxygen and 92% nitrogen for 2 h. The temperature and humidity were maintained at 37°C and 50–80%, respectively. The rats of sham group were treated in the same manner except the ligation of the right carotid artery.

### Zea Longa Scores

As Table 1 shows, the Longa et al. (1989) scores were used to evaluate the nerve function of rats at 0 time point, 6, 12, 24, and 48 h after HI. If the rat had no neurological defect, the score was 0. If the rat could not fully stretch left forepaw, the score was 1. If the rat circled while walking, the score was 2. If the rat tumbled while walking because of hemiplegia, the score was 3. If the rat was unconscious and unable to walk, the score was 4.

**Table 1:** Zea Longa scores.

Zea-Longa score	The performance of rats
0	The rat has no neurological defect
1	The rat is failure to extend left forepaw fully
2	The rat circles while walking
3	The rat tumbles to its side while walking because of hemiplegia
4	The rat is unconscious and unable to walk


### Magnetic Resonance Imaging (MRI)

At 1 month after HI, the cerebral infarction of the rats was demonstrated by MRI. T2WI tests were completed on a 7.0 T Bruker BioSpec scanner with a 75mm diameter volume coil for radio frequency pulse transmission and a quadrature surface coil for signal detection. During the imaging sessions, the animals were anesthetized as previously described. The body temperature of the animals was maintained at 36 ± 1°C using a warm water pad. T2WI anatomical images were acquired with a rapid acquisition with spin echo (SE) sequence, repetition time (TR) = 2961.5 ms, echo time (TE) = 33 ms, field of view (FOV) = 3.5 cm 9 3.5 cm, matrix = 256 9 256, slice thickness = 1.0 mm. The total acquisition time was 10 min.

### Tissue Harvesting

Rats were deeply anesthetized with sevoflurane. After supine fixation, the chest of rats was opened to expose the heart and ascending aorta. Each rat was perfused with 15 ml PBS to remove blood from circulation. Then, rat brains were removed and placed in a Petri dish containing 0.9% normal saline on ice. After stripping the meninx, the cortex and hippocampus were separated carefully. The tissues were then stored at -80°C for further use.

### Quantitative Real-Time PCR (qRT-PCR)

Total RNA was isolated using Trizol (Thermo Fisher Scientific, United States). A TaqMan MicroRNA Reverse Transcription kit (Applied Biosystems, United States) was used to generate cDNA of miR-7a-2-3p. We performed qRT-PCR of miR-7a-2-3p by using All-in-One TM qPCR Mix (GeneCopoeia, Rockville, MD, United States) on an ABI 7500HT System (Applied Biosystems) to determine the expression levels of miRNA-7a-2-3p. The target sequence was amplified using reaction mixtures which included 12.5 μL 2 × PCR Master Mix, 0.5 μL upstream primer, 0.5 μL downstream primer, 10.5 μL PCR Nuclease-Free Water and 1 μL cDNA. U6 and β-Actin were used as endogenous controls, respectively. The primers used in the protocol were as follows: miR-7a-2-3p, 5-CAACAAGTCCCAGTCTGC-3 (forward) and 5-CAGTGATGTTGCGGTCTG-3 (reverse); U6, 5-CTCGCTTCGGCAGCACA-3 (forward) and 5-TGGTGTCGTGGAGTCG-3 (reverse); β-Actin, 5-CTCGCTTCGGCAGCACA-3 (forward) and 5-TGGTGTCGTGGAGTCG-3 (reverse); Vimentin (VIM), 5-CGCTTCGCCAACTACATC-3 (forward) and 5-TCTTCATCGTGCAGCTTCTT-3 (reverse); pleiomorphic adenoma gene 1(PLAG1), 5-CTTCCAGCGAACTCTTGT-3 (forward) and 5-GCCACTCTGAGGCTCTAAC-3 (reverse); dual specificity phosphatase 10 (DUSP10), 5-AATGACCTGGCGAAGAAGAT-3 (forward) and 5-AACTATGTGAAGTGGTTGGGAT-3 (reverse); NAD(P)H dehydrogenase, quinone 1 (NQO1), 5-GCGTCTGGAGACTGTCTGGG-3 (forward) and 5-ATCTGGTTGTCGGCTGGAAT-3 (reverse); tumor necrosis factor receptor superfamily, member 1B (TNFRSF1B), 5-TGGGTCTGCTGATGTTAGG-3 (forward) and 5-CTCCGCTGTGACTCTTGC-3 (reverse). All reactions were performed for 40 cycles, the fluorescence value was recorded from each reaction. The PCR products amplified in the PCR instrument were analyzed by electrophoresis using 1% agarose gel and visualized by GoldView with Alpha Innotech (Bio-Rad, Hercules, CA, United States) to determine if the positive bands were obtained.

### Culture of PC12 Cells and SH-SY5Y Cells

PC12 cells and SH-SY5Y cells were purchased from American Type Culture Collection (ATCC, United States) and cultured according to the guidelines recommended by ATCC. All cell lines were cultured in Dulbecco’s Modified Eagle Medium (DMEM) (×1) (cat. no. 10569-010) supplemented with 10% fetal bovine serum (cat. no. 16000-077). All cells were maintained at 37°C and 5% CO_2_. The number of cells were quantified with Image-Pro Plus 6.0 software (Media Cybernetics, United States) at 24 h after oxygen-glucose deprivation (OGD) induction.

### Culture of Primary Cortical Neurons

Primary cortical neurons were obtained from one-day-postnatal SD rats. Briefly, the cortexes of rats were harvested and cut into approximately 1 mm^3^ small pieces, then digested with 0.05% trypsin (Gibco, Thermo Fisher Scientific, United States) at 37°C for 10 min and eluted with 10% FBS (bovine serum albumin, Gibco, Thermo Fisher Scientific, United States). The tissue suspension was centrifuged at 1000 rpm for 10 min. The pellets were resuspended in complete culture medium (HyClone, United States) composed of DMEM/HIGH GLUCOSE, 10% FBS (Gibco, United States) and 1% penicillin-streptomycin solution (HyClone, United States). Neurons were plated in 96-well plates (Corning, United States) coated with poly lysine and laminin (Sigma-Aldrich, St. Louis, MO, United States) at a density of 5 × 10^5^ cells/ml. After four hours of incubation at 37°C and 5% CO_2_, the complete culture medium was replaced with neurobasal medium with the addition of 2% B27 (Invitrogen, Carlsbad, CA, United States). One-half of the culture medium was changed every 3 days.

### Transfection of miR-7a-2-3p

For the transfection of miR-7a-2-3p, PC12 cells, SH-SY5Y cells and primary cortical neurons, were divided into four groups: normal group, OGD group, OGD+ mimic negative control (miR-NC) group, OGD+miR-7a-2-3p group. MiR-7a-2-3p was designed and synthesized by RiboBio (Guangzhou, China). Transfection was performed using the SuperFectinTM II *in vitro* transfection reagent (Pufei Biotech, China). Briefly, a mix of transfection stock Buffer and miR-7a-2-3p mimic was prepared and 3 μl of SuperFectin^TM^ II reagent was added to the mixture. Mixture of miR-7a-2-3p mimic (50 nM) was added drop-wise to the appropriate wells. After incubation at 37°C for 24 h, another 1.2 ml of fresh culture medium was added and not replaced.

### Construction of Oxygen Glucose Deprivation (OGD) Model *in vitro*

After transfection of miR-7a-2-3p, the model of OGD was established in PC12 cells, SH-SY5Y cells and primary cortical neurons. The culture medium in each cell type was replaced with the medium that had no glucose, and the cells were placed into an incubator with 0.1% oxygen, 95% N_2_, 5% CO_2_, at 37°C. After 2 h, the culture medium was replaced by DMEM medium and neuron specific medium, then the cells were put in the incubator at 37°C and 5% CO_2_.

### Methyl Thiazolyl Tetrazolium (MTT) Analysis

The cell viability of neurons was examined using the MTT assay, which was performed at 24 h after OGD induction. In brief, neurons were seeded in 96-well plates that had been precoated with poly-L-lysine. At 24 h after OGD, neurons were incubated in a serum-free medium containing MTT solution in the dark for 4 h at 37°C. The MTT solution was discarded and 100 μl DMSO was added to each well to dissolve the formazan crystals. The value of optical density was measured at a wavelength of 562 nm using a Bio-Rad microplate reader (Bio-Rad, Hercules, CA, United States). Cell survival was calculated as a percentage relative to the control.

### Tuj1 Staining

Tuj1 staining was performed to detect the changes of neuron morphologies after miR-7a-2-3p transfection. The neurons were incubated at 4°C with the anti-Tuj1 rabbit antibody (1:200, Immunoway, United States) in a moist chamber for 18 h. After being washed three times, the neurons were incubated with fluorescence-labeled goat secondary antibody (1:200, Immunoway, United States) at 37°C for 1 h. The nuclei were stained by DAPI to produce blue color. Subsequently, the neurons were observed under the fluorescence microscopy (Leica, CM1860, Germany).

### TUNEL Assay to Test Cell Apoptosis

TUNEL enzyme and labeling solutions (In Situ Cell Death Detection Kit, TMR red; Cat. NO. 12156792910, Roche, Switzerland) were mixed at a ratio of 1:9 (*v*/*v*) and applied to the neurons at 4°C overnight in the dark. After washing with PBS, the neurons were stained with DAPI for 5 min at room temperature, and photographs were obtained under fluorescence microscopy (Leica, CM1860, Germany). The nuclei of apoptotic neurons were stained in red by TUNEL and the nuclei of neurons were stained in blue by DAPI. Apoptosis was quantified by determining the percentage of TUNEL/DAPI using Image-Pro Plus 6.0 software (Media Cybernetics, United States).

### Forecasting of the Target Genes of miR-7a-2-3p

To further confirm the underlying mechanism of miR-7a-2-3p promoting neurons survival and proliferation, we searched the target genes of miR-7a-2-3p in the miRNA sequencing, mi-RDB, TargetScan, miR-map. Then, the intersection of the predicted target genes of miR-7a-2-3p was acquired. As above shown, the relative expression of forecasted gene was determined by qRT-PCR.

### Statistical Analysis

All data in the experimental process are presented as mean ± standard deviation (SD). Statistical analysis was performed by using SPSS 19.0 software. The comparison between the two groups was analyzed by Student’s *t*-test. When there were three groups and more, data were subjected to a one-way analysis of variance. *P* < 0.05 value was considered to be statistically significant. ^∗^*P* < 0.05, ^∗∗^*P* < 0.01.

## Results

### The Levels of MiR-7a-2-3p Are Decreased in Cerebral Cortex After HIBD *in vivo*

**FIGURE 1:**
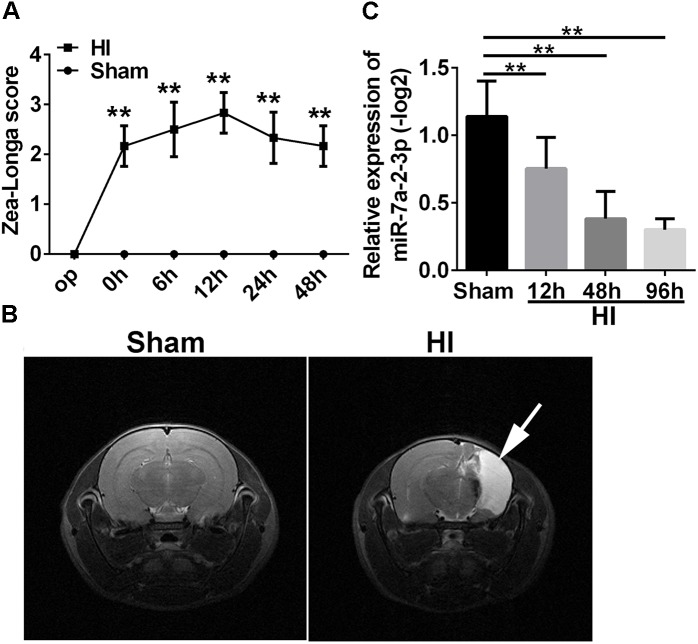
The levels of MiR-7a-2-3p are Decreased in Cerebral Cortex after HIBD *in vivo*. **(A)** The Zea Longa scores were statistically different at 0 time point, 6, 12, 24, and 48 h after HI compared with the sham group (^∗^*^∗^P* < 0.01). **(B)** MRI showed the cerebral ischemia infraction at 1 month after HI. The white arrow shows the area of cerebral ischemia infraction. **(C)** The expression of miR-7a-2-3p was decreased at 12, 48, and 96 h after HI (^∗^*^∗^P* < 0.01).

In order to investigate the mechanisms of neuronal HIBD, we established the HI model in rats which was based on the classical Rice–Vannucci method (Rice et al., 1981). To confirm the successful establishing of HI model, Zea Longa Scores Analysis was employed (Figure 1A). There was a statistically significant difference in the scores at 0 time point, 6, 12, 24, and 48 h after HI when compared with the sham group (*n* = 6, ^∗∗^*P* < 0.01). Meanwhile, there was an obvious recovery at 48 h, but the score was still higher than in the sham group. In addition, the Magnetic Resonance Imaging (MRI) showed that the right brain had significant infarction area in the HI group (Figure 1B) at 1 month after HI, which suggested the successful establishing of the HI model *in vivo*. In order to investigate a potential role of miR-7a-2-3p in neuronal HIBD, we examined changes in miR-7a-2-3p expression after neuronal HIBD by qRT-PCR and have found that miR-7a-2-3p significantly decreased at 12, 48, and 96 h after HI in rats (*n* = 6, Figure 1C).

### The Levels of MiR-7a-2-3p Are Decreased in PC12 Cells, SH-SY5Y Cells and Primary Cortical Neurons After OGD *in vitro*

**FIGURE 2:**
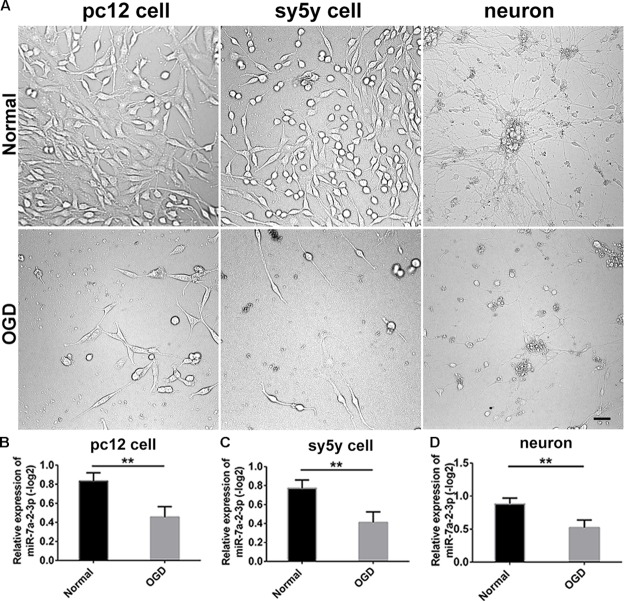
The levels of MiR-7a-2-3p are Decreased in PC12 cells, SH-SY5Y Cells and Primary Cortical Neurons after OGD *in vitro*. **(A)** The images of PC12 cells, SH-SY5Y cells, and neurons after OGD are shown. Bar, 50 μm. **(B)** miR-7a-2-3p was downregulated significantly in PC12 cells after OGD injury when assessed by PCR (*^∗∗^P* < 0.01). **(C)** miR-7a-2-3p was downregulated significantly in SH-SY5Y cells after OGD when assessed by PCR (^∗∗^*P* < 0.01). **(D)** miR-7a-2-3p was downregulated significantly in neurons after OGD when assessed by PCR (^∗∗^*P* < 0.01).

We conducted the OGD model in PC12 cells, SH-SY5Y cells and primary cortical neurons. The bright field images obtained via optical microscopy are presented in Figure 2A. It is evident that the cell number and neurite length were significantly decreased in the OGD model in comparison to the normal group (Figure 2A). We have also found that miR-7a-2-3p was significantly decreased in PC12 cells (*n* = 6), SH-SY5Y cells (*n* = 6) and neurons (*n* = 6) after OGD in the remaining cells (Figures 2B–D).

### Overexpression of MiR-7a-2-3p Improved the Morphology and Increased the Number of PC12 Cells, SH-SY5Y Cells and Primary Cortical Neurons After OGD

**FIGURE 3:**
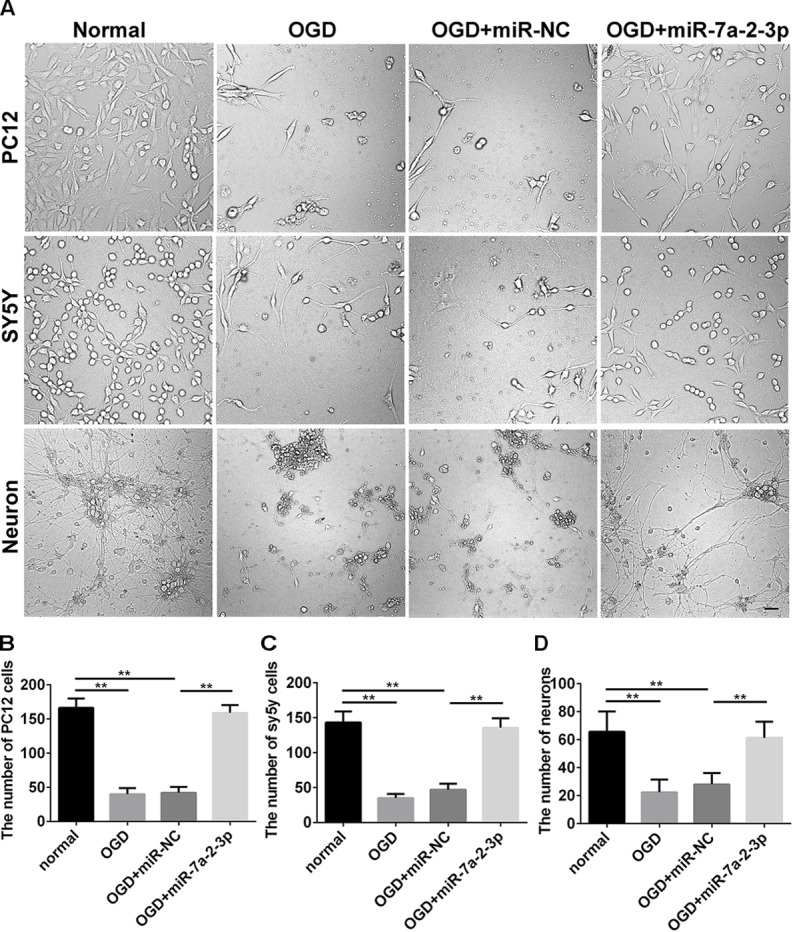
Overexpression of MiR-7a-2-3p Improved the morphology and increased the Number of PC12 cells, SH-SY5Y Cells and primary cortical Neurons after OGD injury. **(A)** The morphological changes of PC12 cells, SH-SY5Y cells and neurons are shown for the normal group, OGD group, OGD+ miR-NC group and OGD+ miR-7a-2-3p group. Bar, 50 μm. **(B)** The number of PC12 cells in OGD+ miR-7a-2-3p group significantly increased compared with the OGD group (*^∗∗^P* < 0.01). **(C)** The number of SH-SY5Y cells in OGD+ miR-7a-2-3p group significantly increased compared with the OGD group (*^∗∗^P* < 0.01). **(D)** The number of neurons in OGD+ miR-7a-2-3p group significant increased compared with the OGD group (*^∗∗^P* < 0.01).

In the study, we found the expression of miR-7a-2-3p was increased after transfection of miR-7a-2-3p mimic (Supplementary Figure S1). The images of PC12 cells, SH-SY5Y cells and neurons are shown for the normal group, OGD group, OGD+miR-NC group, OGD+miR-7a-2-3p group (Figure 3A). We have found that the number of PC12 cells, SH-SY5Y cells and neurons was decreased significantly after OGD versus normal groups (*n* = 6, ^∗∗^*P* < 0.01) (Figures 3B–D). However, overexpression of miR-7a-2-3p promoted restoration of the cell numbers (Figures 3B–D) and also, improved the cell morphology (Figure 3A) of all three cell types after OGD (*n* = 6, ^∗∗^*P* < 0.01). In conclusion, we found that miR-7a-2-3p could ameliorate injury of cells *in vitro* caused by OGD.

### Overexpression of MiR-7a-2-3p Ameliorated Neurodegeneration and Improved the Growth of Neuronal Axons

**FIGURE 4:**
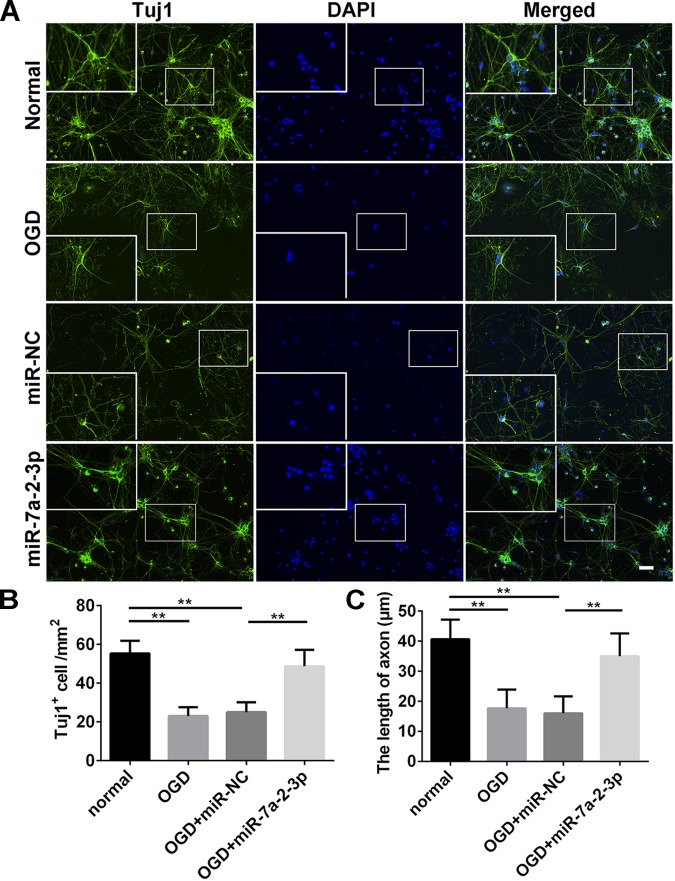
Overexpression of MiR-7a-2-3p Ameliorated Neurodegeneration and improved the Growth of Neuronal Axons. **(A)** Tuj1^+^ neurons (green) are shown for the normal group, OGD group, OGD+miR-NC group and OGD+miR-7a-2-3p group. Nuclei were visualized by DAPI (blue). Bar, 50 μm. **(B)** The number of Tuj1^+^ cells was increased in OGD+miR-7a-2-3p group compared with the OGD+miR-NC (*^∗∗^P* < 0.01). **(C)** The axonal length of neurons was increased in the OGD+miR-7a-2-3p group compared with the OGD+miR-NC group (*^∗∗^P* < 0.01).

Tuj1+ cells were investigated in the normal group, OGD group, OGD+miR-NC group and OGD+miR-7a-2-3p group (Figure 4A). The number of Tuj1+ cells was significantly reduced in both the OGD group and miR-NC group, when compared with the normal group. However, after transfection of miR-7a-2-3p, the number of Tuj1+ cells was significantly increased in the OGD+miR-7a-2-3p group, versus the OGD+miR-NC group (*n* = 6, ^∗∗^*P* < 0.01) (Figure 4B). The length of neuronal axons was significantly reduced in both the OGD group and miR-NC group compared with the normal group, however, the average axonal length was significantly increased after miR-7a-2-3p transfection (*n* = 6, ^∗∗^*P* < 0.01) (Figure 4C). In a nutshell, Tuj1 staining showed that overexpression of miR-7a-2-3p could improve the morphological changes of neurons, as well as the cellular number and axonal length after OGD injury.

### Overexpression of MiR-7a-2-3p Reduced the Apoptosis of Neurons After OGD

**FIGURE 5:**
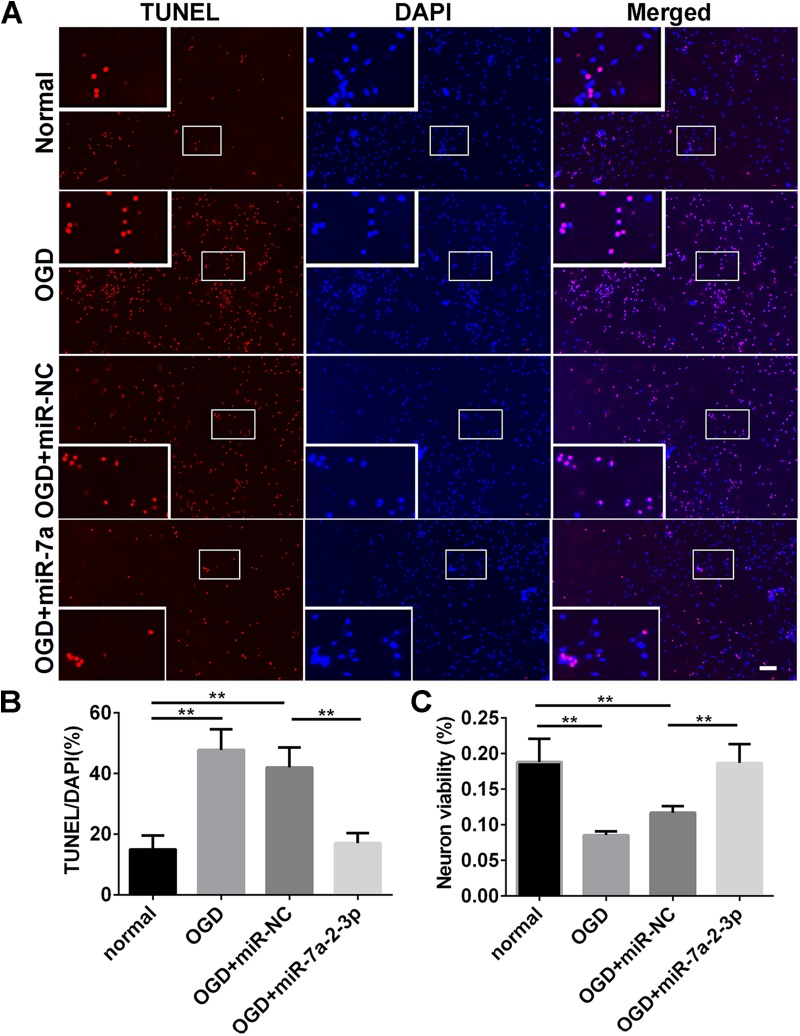
Overexpression of MiR-7a-2-3p Reduced the Apoptosis of Neurons after OGD injury. **(A)** The apoptotic changes in neurons (red) are shown for the normal group, OGD group, OGD+miR-NC group and OGD+ miR-7a-2-3p group. Nuclei were visualized by DAPI (blue). Bar, 50 μm. **(B)** The apoptotic ratio of neurons significantly increased in the OGD group compared with the normal group. When compared with the OGD+miR-NC group, the apoptotic ratio of neurons significantly decreased in the OGD+miR-7a-2-3p group (^∗∗^*P* < 0.01). **(C)** The viability of neurons was quantified by MTT. The viability of neurons was markedly decreased in the OGD group compared with the normal group. When compared with the OGD+miR-NC, the viability of neurons significantly increased in the OGD + miR-7a-2-3p group (^∗∗^*P* < 0.01).

In order to explore the effect of miR-7a-2-3p overexpression on the neuronal apoptosis, we performed TUNEL and MTT assays in neurons. The apoptotic cells were investigated in the normal group, OGD group, OGD+ miR-NC group and OGD+ miR-7a-2-3p group (Figure 5A). In the OGD and OGD + miR-NC groups, the neuronal apoptosis was increased compared with the normal group. However, the neuronal apoptosis was significantly decreased after transfection of cells with miR-7a-2-3p (*n* = 6, ^∗∗^*P* < 0.01) (Figure 5B). The neuron viability was also assessed by the MTT assay, and the neurons viability was increased after transfecting miR-7a-2-3p in the OGD model (*n* = 6, ^∗∗^*P* < 0.01) (Figure 5C). These results suggest that miR-7a-2-3p contributed to the decreased neuronal apoptosis and increased neuronal viability after OGD injury.

### Target Gene Prediction of miRNA-7a-2-3p

**FIGURE 6:**
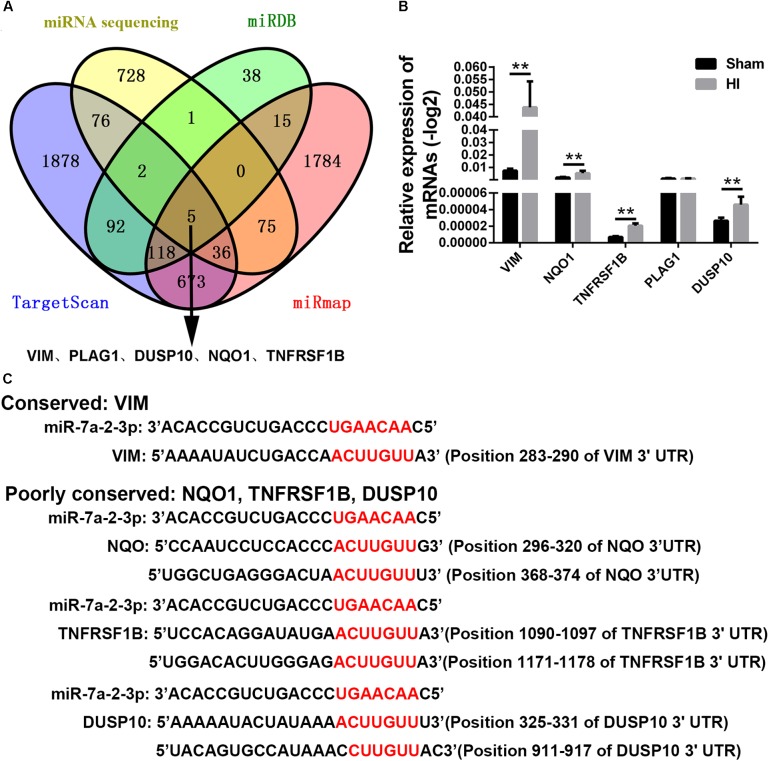
Target Gene Prediction of miRNA-7a-2-3p. **(A)** The intersections of the predicted target genes results of miR-7a-2-3p in TargetScan, miRDB, miRwalk and miRNA sequencing are presented. The different colors represent distinctive methods of prediction. There were five target genes of miR-7a-2-3p, including VIM, PLAG1, DUSP10, NQO1, and TNFRSF1B. **(B)** The qRT-PCR analysis showed that VIM, PLAG1, DUSP10, NQO1, and TNFRSF1B. VIM, PLAG1, DUSP10, NQO1, and TNFRSF1B were significantly increased in the HI group compared with the sham group (*^∗∗^P* < 0.01). **(C)** Alignment of the seed regions of miR-7a-2-3p with 3’-UTR of VIM, PLAG1, DUSP10, NQO1, and TNFRSF1B is shown. The red color indicated the pairing bases.

In order to find the target genes of miRNA-7a-2-3p, we took intersection of the predicted target genes results of miR-7a-2-3p in TargetScan, miRDB, miRwalk, and mRNA sequencing by the bioinformatics prediction. We found five potential target genes of miR-7a-2-3p, such as VIM, PLAG1, DUSP10, NQO1 and TNFRSF1B (Figure 6A). Moreover, we determined the relative expression of those genes through qRT-PCR. There were four target genes (VIM, DUSP10, NQO1, and TNFRSF1B) which were markedly increased in the HI group compared with the sham group (*n* = 6, *^∗∗^P* < 0.01) (Figure 6B). The alignment of the seed regions of miR-7a-2-3p with 3’-UTR of VIM, DUSP10, NQO1, and TNFRSF1B are shown in Figure 6. We have also found that VIM was widely conserved and DUSP10, NQO1 and TNFRSF1B were poorly conserved across the species (Figure 6C).

## Discussion

In our study, we confirmed that the expression of miR-7a-2-3p is decreased in the neonatal HIBD, whereas its overexpression could promote neuronal survival and reduce neuronal apoptosis. In addition, this study indicated that VIM might be the action target gene of miR-7a-2-3p. The present study suggested that miR-7a-2-3p might play a crucial role in neonatal HIBD and VIM might be its direct target gene, thus providing a guide for future fundamental research and possible clinical therapeutic strategy.

In this study, we established the HI model *in vivo* and OGD model *in vitro* and found that the levels of miR-7a-2-3p are significantly decreased in hypoxic-ischemic damage. The HI model was examined by Zea Longa scores (Chandra et al., 1999; Legos et al., 2008; Lenhard et al., 2008) which were used to verify the degree of neurologic deficits (Maguire et al., 2004; Ashwal et al., 2006, 2007). The cerebrum infarction was evident according to MRI at 1 month in the HI rats, also confirming that HI model was successfully established. Then, we obtained the cortical tissues of rats at 12, 48, and 96 h after HI to investigate the expression of miR-7a-2-3p by qRT-PCR, which showed that the levels of miR-7a-2-3p were significantly decreased after hypoxic-ischemic brain injury. In the previous study, researchers have found that miR-124-3p, the most brain-enriched miRNA, was significantly reduced after cerebral ischemia and hypoxia in stroke. They have also found that miR-124-3p was SVZ neuronal fate determinant and mediated stroke-induced neurogenesis in adult SVZ and striatum (Liu et al., 2011; Akerblom et al., 2012; Doeppner et al., 2013). Other researchers have found that miR-207 and miR-352 were mainly down-regulated in rats after the middle cerebral artery occlusion, and played a role in regulating neuronal apoptosis. Hereby, we hypothesized that miR-7a-2-3p might play a significant role in neonatal HIBD.

In present study, we have found that overexpression of miR-7a-2-3p promoted neuronal survival and reduced neuronal apoptosis after OGD, which suggested that it might be the key regulated target site in ischemic injury. Previous studies have shown that miR-7 could suppress secretion of insulin, indicating that it might become a new target for improving β cell function in diabetes (Xu et al., 2015). Other researchers have also reported that miR-7a-2-3p was responsible for protecting cardiac myocytes against hypoxia-induced apoptosis (Geng et al., 2016). It also could significantly influence self-renewal ability of Embryonic stem cells (ESCs) by targeting Klf4 (Wang et al., 2016). Further study proved that miR-7 targeted Herpud2 3’UTR, which encoded endoplasmic reticulum (ER) stress protein-HERP2. MiR-7 depressed the ER-related protein expression including glucose-regulated protein 78 (GRP78), C/EBP-homologous protein (CHOP), inhibited pro-inflammatory cytokines including tumor necrosis factor α (TNF-α), and thereby attenuated the OGD-mediated inflammatory response and astrocytic damage (Dong et al., 2016). In other studies, hypoxia has been validated to modulate miRNAs’ expression (Nallamshetty et al., 2013). Our findings demonstrated that miR-7a-2-3p could suppress neuron apoptosis and promoted neuron survival after OGD injury. Furthermore, we predicted the target gene of miR-7a-2-3p. Our findings showed that VIM contained a certain conserved sequence complementary with miR-7a-2-3p, indicating that it might play a key role in the neuroprotection. The expression of miR-7a-2-3p and VIM demonstrated a negative correlation between miR-7a-2-3p and VIM, which indicated that VIM might be the direct target gene of miR-7a-2-3p in hypoxic-ischemic neuron injury.

## Conclusion

Taken together, miR-7a-2-3p might be an important regulatory molecule of apoptosis after hypoxic-ischemic neuron damage. Moreover, as the target gene of miR-7a-2-3p, VIM might have a role in hypoxic-ischemic neuron injury. This study provides new knowledge on the pathogenesis and possible treatments of cerebral ischemia, while detailed molecular mechanisms still need to be further explored.

## Author Contributions

T-HW conceived and designed the experiments. Z-BZ, Y-XT, QZ, L-LX, F-FX, and YX performed the experiments, analyzed the data, and wrote the manuscript. JL, LB and X-FZ critically evaluated the manuscript and wrote some parts of the manuscript.

## Conflict of Interest Statement

The authors declare that the research was conducted in the absence of any commercial or financial relationships that could be construed as a potential conflict of interest. The reviewer AV and handling Editor declared their shared affiliation at the time of the review.

## Supplementary Material

FIGURE S1 The expression of miR-7a-2-3p is upregulated after transfection of miR-7a-2-3p in PC12 cells, SH-SY5Y cells and neurons. **(A)** MiR-7a-2-3p was upregulated significantly after transfection of miR-7a-2-3p in PC12 cells (*^∗∗^p* < 0.01). **(B)** MiR-7a-2-3p was upregulated significantly after transfection of miR-7a-2-3p in SH-SY5Y cells (^∗∗^*P* < 0.01). **(C)** MiR-7a-2-3p was upregulated significantly after transfection of miR-7a-2-3p in neuron (^∗∗^*P* < 0.01).
