# Supplementary figures and images for: miRNA-7a-2-3p Inhibits Neuronal Apoptosis in Oxygen-Glucose Deprivation (OGD) Model
Source: Front Neurosci. 2019 Jan 23;13:16. doi: 10.3389/fnins.2019.00016 (PMC6351497; doi:10.3389/fnins.2019.00016)

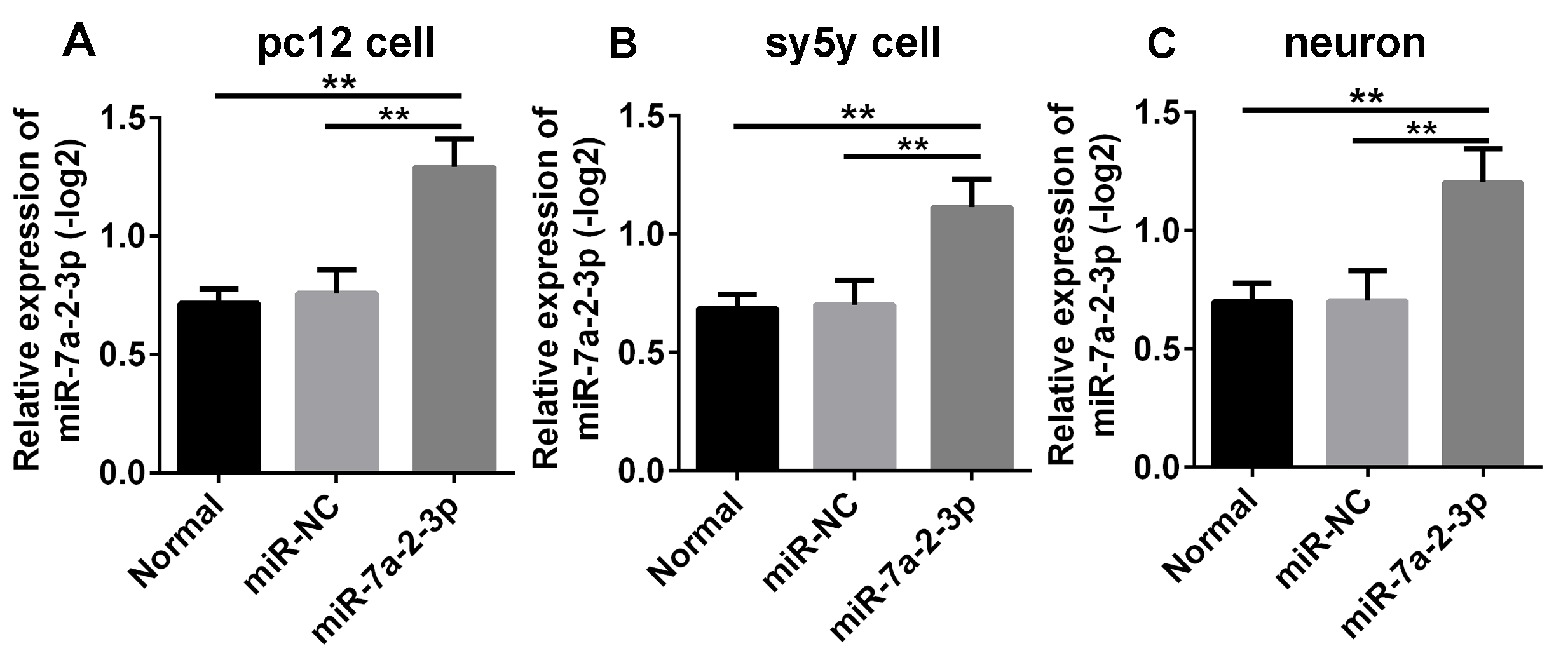

Supplement: FIGURE S1 — The expression of miR-7a-2-3p is upregulated after transfection of miR-7a-2-3p in PC12 cells, SH-SY5Y cells and neurons. (A) MiR-7a-2-3p was upregulated significantly after transfection of miR-7a-2-3p in PC12 cells (∗∗p < 0.01). (B) MiR-7a-2-3p was upregulated significantly after transfection of miR-7a-2-3p in SH-SY5Y cells (∗∗P < 0.01). (C) MiR-7a-2-3p was upregulated significantly after transfection of miR-7a-2-3p in neuron (∗∗P < 0.01). [file Image_1.TIF]
